# Compartment syndrome of the thigh. A case report with delayed onset after stable pelvic ring fracture and chronic anticoagulation therapie

**DOI:** 10.1186/1471-2318-10-51

**Published:** 2010-07-27

**Authors:** Rolf D Burghardt, Sebastian Siebenlist, Stefan Döbele, Martin Lucke, Ulrich Stöckle

**Affiliations:** 1 Department of Trauma Surgery, Klinikum rechts der Isar, Technische Universitaet Muenchen, Muenchen, Germany

## Abstract

Compartment syndrome of the thigh is a rare occurrence potentially leading to devastating functional restrictions. There is a wide spectrum of reported conditions leading to increased tissue pressure in the thigh possibly resulting in a compartment syndrome, ranging from deep venous thrombosis to blunt injuries and femoral fractures. We report a case of a delayed development of a compartment syndrome of the thigh secondary to an undisplaced anterior pelvic ring fracture and chronic anticoagulation therapy in a 94-year-old woman. Regarding anticoagulation therapy there are numerous reports about the spectrum of bleeding complications during therapy, however this severe complication has to our knowledge not been reported previously. Treatment consisted in immediate fasciotomy and subsequently secondary wound closure.

## Background

Blunt trauma or femoral fractures, frequently lead to soft tissue damage and contusion of varying degrees of severity. In comparison to the lower leg the development of a compartment syndrome in the thigh however is particularly rare. In the lower leg the muscle compartments are tighter enclosed by the muscle fascia as in the thigh and the lower dilatability of this muscle fascia promotes the process of the development of a compartment syndrome. As the compartments of the thigh are larger in space and more compliant, they are more sustainable against expanding haematoma [1]. If the intra-compartmental pressure increases and exceeds the perfusion pressure, the microcirculation becomes oppressed and the tissue viability is jeopardized. Volkmann in 1881 [2] was the first to describe these pathophysiologically coherences.

Compartment syndromes of the thigh are rare but they bear the potential danger of serious morbidity and potential mortality if not recognized early and treated immediately. Operative treatment includes immediate fasciotomy of the affected compartments, complete drainage of the haematoma, careful arrest of bleeding, and delayed secondary closure of the skin.

As the compartment syndrome of the thigh is a particular rare occurrence, most articles in the literature report about specific cases [1,3-9], or present small case series [10], screening protocols [11], functional outcome [12] or a clinical spectrum [10,13]. To our knowledge a case of a delayed development of an acute compartment syndrome of the thigh after a stable pelvic ring fracture and chronic anticoagulation therapy has not been reported previously.

## Case Presentation

**Figure 1:**
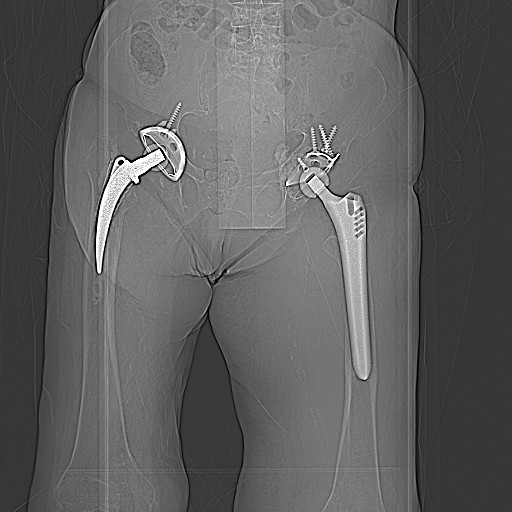
**CT-scout-view showing the nondisplaced anterior pelvic ring fracture on the left side**.

**Figure 2:**
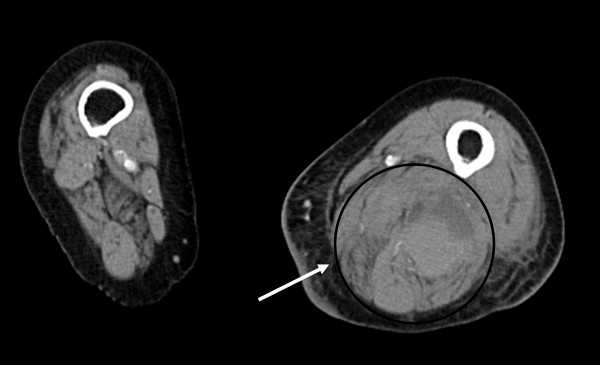
**CT-scan shows in the axial view the huge haematoma formation in the medial and posterior compartment of the thigh**. (Compare the anatomic structures with the schematic in figure 6).

**Figure 3:**
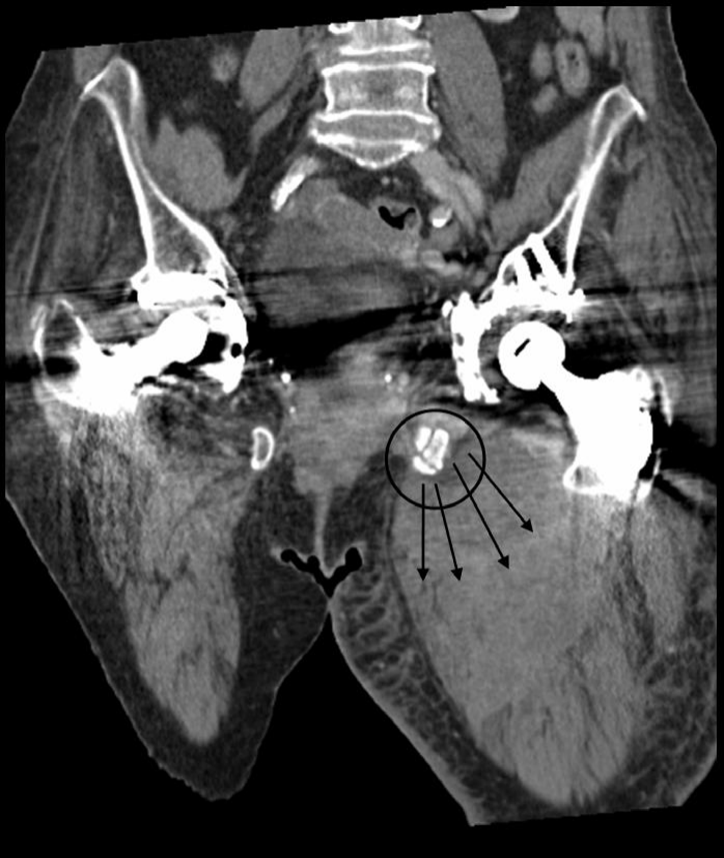
**A & B: CT-scans show in the coronar view the fractures of the left anterior pelvic ring as origin of the bleeding liable for the development of the cpompartment syndrome of the left thigh**.

**Figure 4:**
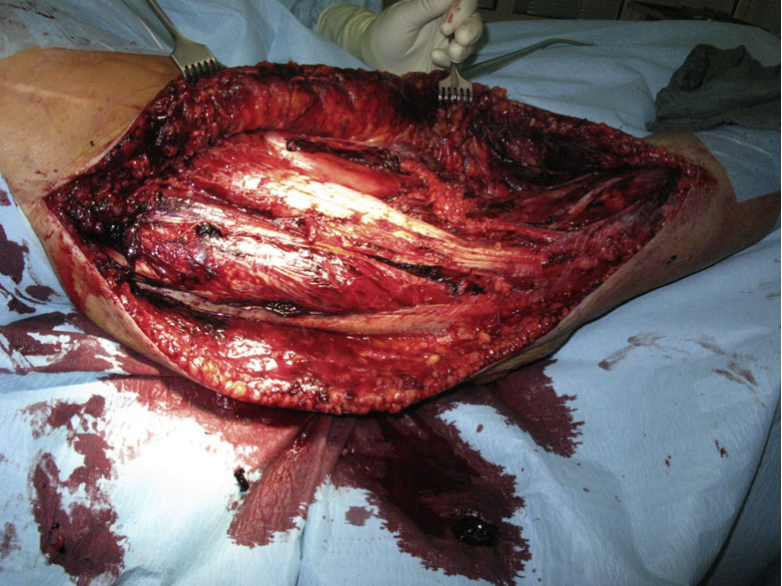
**Clinical picture after complete decompression of the thigh muscle compartments**.

**Figure 5:**
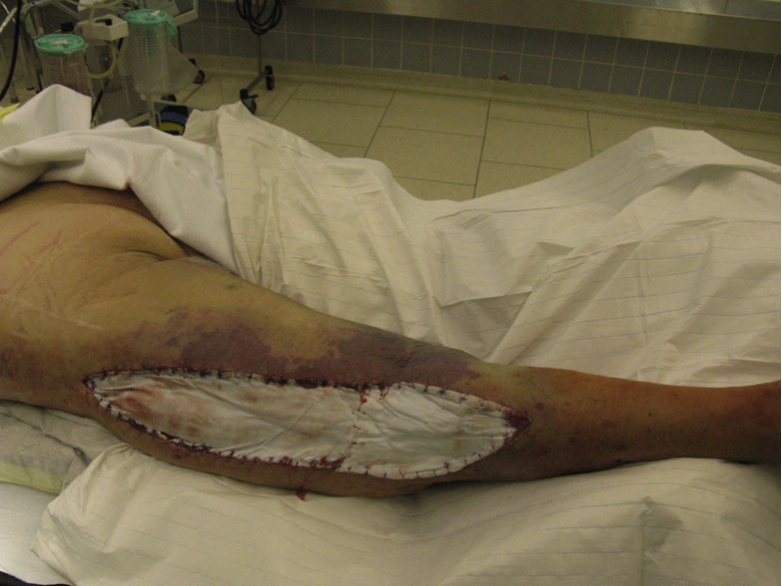
**After draining of the haematoma, wound covering with epigard was performed**.

A 94-year-old female was admitted to our hospital after she fell and sustained an anterior pelvic ring fracture (Figure 1). She was hospitalized for mobilization. Over the past 18 years she had been treated with anticoagulation therapy with marcumar due to atrial fibrillation, 13 years ago subsequently a cardiac pacemaker was implanted. In the first days in hospital mobilisation was painful but with a walker possible. In the early morning of the fourth day after admission she developed severe pain in the thigh. Clinically the thigh was swollen but the foot pulses were well palpable. Because of persisting severe pain and the suspicion of a deep vein thrombosis a CT-scan of the thigh and the pelvis was performed. In the further course the patient complained of a partial loss of sensation of her left leg and weakness of her shank muscles, following the innervation area of the ishiadic nerve. The CT-scan showed an extensive haematoma expanding abroad the adductor muscles in the medial compartment and in the posterior compartment (Figure 2 &3). The compartment of the M. rectus femoris was not involved. Immediate surgery was performed with a complete decompression of the compartments of the thigh as well as the draining of the haematoma and wound covering with epigard (Othomed, Vienna, Austria) (Figure 4 &5). On the second postoperative day a revision for draining of the refilled haematoma was performed. Finally five days after the initial treatment during the second revision complete skin-closure was performed strainless. In the postoperative course the patient recovered quickly and the sensomotor deficits resolved completely however she developed partial skin necrosis along the approach in particular in the popliteal fossa. Subsequently these necrosis were resected and the skin was closed again strainless. Only in the popliteal foassa a skin graft from the ipsilateral posterior thigh was needed. The skin graft healed. In the further course of the hospital stay the patient again recovered quickly and was mobilized under physiotherapy control. Fifty-seven days after the initial trauma the patient was dismissed and transferred to rehabilitation center for further mobilization. At the latest follow up one year post operatively the patient is mobile with full weight-bearing and regular soft tissue condition.

**Figure 6:**
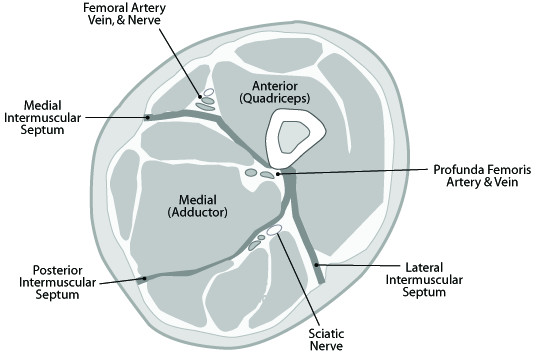
**Cross-sectional anatomy of the thigh, demonstrating the anterior (quadriceps), posterior (hamstrings), and medial (adductor) compartments**. Note the relatiosnship between the intermuscular septa and the neurovascular structures of each compartment.

A full-blown compartment syndrome in the thigh is a rare clinical occurrence. This is mainly due to the fact that the three muscle compartments (Figure 6) in the thigh can compensate much higher volumes than the four compartments below the knee [1]. Not only the fascia seems to be more dilative also the thigh compartments are partly open to the pelvis explaining the higher compensation rate for increasing intracompartmental volume [1,10]. The small number of occurring cases makes scientific randomized prospective studies with bigger numbers difficult. However the spectrum of reported cases with different etiologies is wide: Deep venous thrombosis [7], vein catheriztion [3], vessel aneurysm [6] the complete spectrum of femoral fractures [10], total hip and knee replacement surgery, intramedullary nailing [1,10], gunshot and stab wounds [5], sport trauma [4,8,14-16], traffic accidents [1,10,13], external compression of the thigh [10,17], crush injuries [5,10], as well as severe blunt tissue trauma [10]. Mithoefer [13] declares that in 46% of the cases a traffic accident is the cause for a thigh compartment syndrome. Holbein [1] proclaims that regarding the literature the femur fracture is the most common cause for a thigh compartment syndrome. Kladny [18] numbers this risk with 1-2%. An open fracture or wound will not safely secure the leg from a compartment syndrome [5,10]. Multiple trauma patients seem to have a higher risk to develope a compartment syndrome because of the concurrence of different clinical conditions. High-energy blunt trauma, external compression, systemic hypotension, vascular injury, and coagulopathy in those patients may assimilate and thus lead to a compartment syndrome [10]. According to Schwartz a patient with an isolated injury to the thigh without any of the above mentioned risk factors has therefore a lower risk for the development of a compartment syndrome [10].

In cases were the patient is unconscious at the intensive care unit a compartment syndrome can stay unrecognized possibly causing devastating outcome [1], as the patient can not express main symptoms like disproportional pain, paraesthesia or even paralysis. Only the palpatoric tension of the affected muscle is detectable. These cases must be taken into special account and the measurement of the compartment pressure has an important role in those cases for the diagnostic. Regarding the common literature addressing this topic the measurement of the intracompartmental pressure is the gold standard for the diagnosis [16]. However the reliability of these measurements is questionable, as especially in multiple traumatized patients the systolic blood pressure supported by catecholamines can pretend a stable hemodynamic situation neglecting a possibly severe disregulated microcirculation [19]. In addition there is no consensus in the literature about a specific intracompartmental point pressure or a rule regarding the diastolic blood pressure or the arterial middle pressure, clearly indicating the need of fasciotomy [1]. In our case we initially suspected a deep vein thrombosis and not a compartment syndrome. Therefore we decided for a CT scan. After the diagnosis surgery was performed immediatly and no further diagnostic was performed. Usually suspecting a compartment syndrome calculating the local perfusion pressure by measuring the the mean arterial pressure and the intramascular pressure is the gold standard.

Because of the wide variety of conditions in patients with huge differences in the physiological strength (young men in a car accident versus a 94-year-old women with a stable pelvic fracture) and the very limited numbers of cases with a compartment syndrome of the thigh explains why it is impossible to identify strict criteria for the need of a fascitomy. However the literature agrees that the damage caused by fasciotomy in a patient in which the tissue would not have become necrotic is far outweighed by the morbidity possibly associated by a full-blown compartment syndrome [10]. In a borderline compartment syndrome not only the intracompartmental pressure but close meshed checks of the sensormotor function of the affected leg as well as the typical clinical symptoms are essential for the indication of a fasciotomy.

In our patient the compartment syndrome developed with several days delay. This we hypothezised might be explained by the patient's clinical course. During the first days the patient was immobilized with bed rest. During remobilisation the vessels injured by the initial trauma started to bleed again, thereby creating a haematoma which followed gravity into the thigh. In our case the neurological symptoms resolved quickly after the decompression and no muscle necrosis were detected. However in this case the morbidity was caused by the high age of the patient resulting in multiple complications through a prolonged wound healing. Schwartz has already emphasized that infection is a further problem in the postoperative course in patients with a compartment syndrome. He reports local infection rate of 66% [10]. Although we were not able to detect any significant tissue necrosis in our patient we suppose that prolonged ischemia may have further compromised the microvascular perfusion in this old patient with already existing vascular sclerosis thus drastically increasing the risk of wound infection.

## Conclusion

We observe bleeding complications associated with antikoagulative therapy, the literature presents only few cases in this context. As an yet unreported complication of a compartment syndrome of the thigh secondary to an undisplaced anterior pelvic ring fracture and chronic anticoagulation therapy, we feel this case an important contribution to the literature.

## Competing interests

The authors declare that they have no competing interests.

## Consent

Written informed consent was obtained from the patient for publication of this case report and any accompanying images. A copy of the written consent is available for review by the Editor-in-Chief of this journal.

## Authors' contributions

All authors contributed in a significant way in the steps of processing the patient history as well as writing and editing the manuscript. RDB conceived of the idea for the study and engaged in writing the first draft. SS and SD additional provided expertise in art work. ML and provided research support and advice throughout the project. The senior author US carried out the inevitable surgical procedures and provided geriatric expertise. All authors read and approved the final manuscript.

## Pre-publication history

The pre-publication history for this paper can be accessed here:

http://www.biomedcentral.com/1471-2318/10/51/prepub
